# Kynurenine Promotes RANKL-Induced Osteoclastogenesis In Vitro by Activating the Aryl Hydrocarbon Receptor Pathway

**DOI:** 10.3390/ijms21217931

**Published:** 2020-10-26

**Authors:** Nada H. Eisa, Sakamuri V. Reddy, Ahmed M. Elmansi, Galina Kondrikova, Dmitry Kondrikov, Xing-Ming Shi, Chad M. Novince, Mark W. Hamrick, Meghan E. McGee-Lawrence, Carlos M. Isales, Sadanand Fulzele, William D. Hill

**Affiliations:** 1 Department of Pathology and Laboratory Medicine, Medical University of South Carolina, Charleston, SC 29403, USA; eisa@musc.edu (N.H.E.); elmansi@musc.edu (A.M.E.); kondrikg@musc.edu (G.K.); kondriko@musc.edu (D.K.); 2 Ralph H. Johnson Veterans Affairs Medical Center, Charleston, SC 29403, USA; 3 Department of Biochemistry, Faculty of Pharmacy, Mansoura University, Mansoura 35516, Egypt; 4 Darby Children’s Research Institute, Department of Pediatrics, Medical University of South Carolina, Charleston, SC 29425, USA; reddysv@musc.edu; 5 Department of Neuroscience and Regenerative Medicine, Medical College of Georgia, Augusta University, Augusta, GA 30912, USA; xshi@augusta.edu; 6 Department of Orthopaedic Surgery, Medical College of Georgia, Augusta University, Augusta, GA 30912, USA; MHAMRICK@augusta.edu (M.W.H.); MMCGEELAWRENCE@augusta.edu (M.E.M.-L.); CISALES@augusta.edu (C.M.I.); SFULZELE@augusta.edu (S.F.); 7 Department of Oral Health Sciences, College of Dental Medicine, Medical University of South Carolina, Charleston, SC 29425, USA; novincec@musc.edu; 8 Department of Stomatology, College of Dental Medicine, Medical University of South Carolina, Charleston, SC 29425, USA; 9 Center for Healthy Aging, Medical College of Georgia, Augusta University, Augusta, GA 30912, USA; 10 Cellular Biology and Anatomy, Medical College of Georgia, Augusta University, Augusta, GA 30912, USA; 11 Department of Medicine, Medical College of Georgia, Augusta University, Augusta, GA 30912, USA; 12 Division of Endocrinology, Diabetes and Metabolism, Medical College of Georgia, Augusta University, Augusta, GA 30912, USA

**Keywords:** Kynurenine, AhR, osteoclast, NFATc1, c-fos

## Abstract

There is increasing evidence of the involvement of the tryptophan metabolite kynurenine (KYN) in disrupting osteogenesis and contributing to aging-related bone loss. Here, we show that KYN has an effect on bone resorption by increasing osteoclastogenesis. We have previously reported that in vivo treatment with KYN significantly increased osteoclast number lining bone surfaces. Here, we report the direct effect of KYN on receptor activator of nuclear factor kappa-B ligand (RANKL)-induced osteoclastogenesis in Raw 264.7 macrophage cells, and we propose a potential mechanism for these KYN-mediated effects. We show that KYN/RANKL treatment results in enhancement of RANKL-induced osteoclast differentiation. KYN drives upregulation and activation of the key osteoclast transcription factors, c-fos and NFATc1 resulting in an increase in the number of multinucleated TRAP+ osteoclasts, and in hydroxyapatite bone resorptive activity. Mechanistically, the KYN receptor, aryl hydrocarbon receptor (AhR), plays an important role in the induction of osteoclastogenesis. We show that blocking AhR signaling using an AhR antagonist, or AhR siRNA, downregulates the KYN/RANKL-mediated increase in c-fos and NFATc1 and inhibits the formation of multinucleated TRAP + osteoclasts. Altogether, this work highlights that the novelty of the KYN and AhR pathways might have a potential role in helping to regulate osteoclast function with age and supports pursuing additional research to determine if they are potential therapeutic targets for the prevention or treatment of osteoporosis.

## 1. Introduction

Bone is a unique and vital organ in vertebrates. It plays an important role in movement, hematopoiesis, and storage of minerals. With aging, there is a sharp decrease in the regenerative capacity of the skeletal tissue, which leads to a higher incidence of bone loss, osteoporosis and hip fractures, as well as other age-related bone ailments [1,2]. This can be explained in part by the age-associated decline in osteogenic differentiation of bone marrow mesenchymal stem cells (BMSCs) resulting in less bone formation [3]. However, alongside this, there is also a corresponding increase in osteoclast-mediated bone resorption with aging. The combination of decreased bone formation coupled with increased bone resorption is a hallmark of osteoporosis and dealing simultaneously with both of these actors has been a major challenge in addressing osteoporosis therapeutically. Here, we show a novel mechanism that appears to regulate both processes and presents a unique opportunity to develop a balanced therapeutic approach to age-related bone loss and osteoporosis.

Receptor activator of NF-κB ligand (RANKL) expression levels are increased with aging in both stromal/osteoblast progenitors and osteoclast progenitors [4,5]. The results of these age-related changes in bone homeostasis are an overall decline in bone mass and higher susceptibility to osteoporotic bone fractures. Hence, studying the underlying mechanisms of such changes is essential in both prevention and treatment of osteoporosis. Recently, the kynurenine (KYN) pathway has been gaining increased research focus in osteoporosis and other aging-related conditions. Kynurenine, as well as many of its metabolites, play roles in inflammation, immunity, and cancer [6,7,8,9,10]. Our group has shown that KYN, a tryptophan metabolite that accumulates as we age, induces bone loss in mice by means of impairing osteoblast differentiation and upregulating bone resorption [11]. We dissected the mechanism of KYN’s inhibitory effect on osteogenic differentiation and found that it was mediated via Aryl Hydrocarbon Receptor by upregulating the pro-aging miRNA miR-29b-1-5p and downregulating the chemokine CXCL12 and the epigenetic regulator Histone Deacetylase 3 [12]. Tryptophan is an essential amino acid that can be oxidized to KYN, the first stable tryptophan metabolite, by exposure to reactive oxygen species, as well as by the enzymes tryptophan 2,3-dioxygenase (TDO), found in the liver, and indoleamine 2,3-dioxygenase-1 and 2 (IDO-1 or IDO-2), found in the periphery—including musculoskeletal tissues [12]. We also showed that KYN induced senescence and inhibited autophagy again via the AhR signaling pathway in BMSCs [13]. Additionally, with our group Kim et al., using human bone marrow aspirates showed that increased bone fragility along with TRAP-5b and RANKL levels during aging were associated with higher bone marrow KYN levels [14]. However, no in vitro or in vivo studies have demonstrated the mechanism by which KYN affects bone resorption.

The aryl hydrocarbon receptor (AhR) is the only known receptor for KYN [15,16]. While the role of KYN on osteoclast differentiation has not been thoroughly studied, the involvement of AhR signaling in bone homeostasis have been established, with most of the literature pointing to AhR as a negative regulator of bone mass. Work by Iqbal et al., shows that two smoke toxins, benzo(a)pyrene (BaP) and 2,3,7,8-tetrachlorodibenzo-p-dioxin (TCDD), act as AhR agonists to induce osteoclastic bone resorption in mice by activating cytochrome P450 1a/1b (Cyp1A/1B) enzymes [17]. Other work by Yu et al., shows similar results using 3-methylcholanthrene, a different AhR agonist [18]. They also reported that AhR knockout mice have higher bone mass with a decrease in both bone resorption and formation [18]. Other work by the same group demonstrates that 3,3′-Diindolylmethane, a natural AhR antagonist, suppresses osteoclastic bone resorption and increases bone mass in mice [19]. Interestingly, research from Voronov et al., and Jia et al., show that BaP and tetrandrine, respectively, inhibit Raw 264.7 cells differentiation into osteoclasts via AhR signaling, which contradicts earlier reports [20,21]. This suggests that the AhR signaling effect on osteoclastogenesis may vary according to the specific ligand used, the effect of duration of AhR-ligand binding, the co-factors that interact with the complex and the resulting downstream signaling cascade initiated and ultimately, the specific genes targeted as a result of AhR-ligand binding [22,23].

The present work aims to investigate the effect of KYN on osteoclast differentiation, proliferation, and activity, as well as elucidate the mechanism underlying KYN’s effects.

## 2. Results

### 2.1. Kynurenine Treatment Enhances and Drives RANKL-Mediated Osteoclast Differentiation of Raw 264.7 Cells

**Figure 1:**
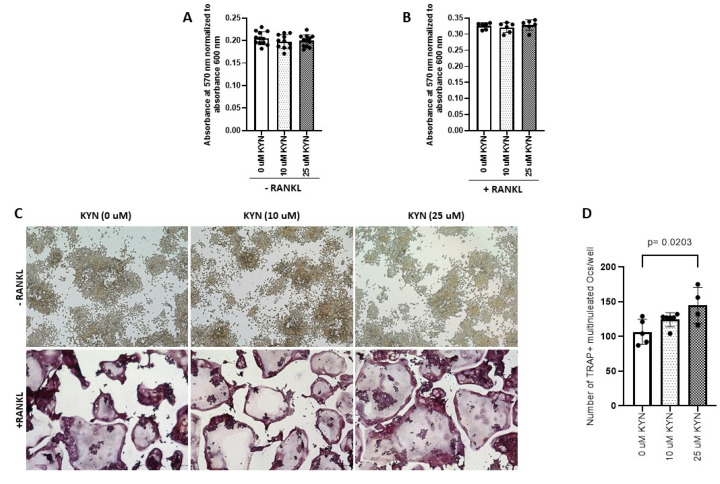
Kynurenine (KYN) treatment induces RANKL-mediated osteoclast differentiation of Raw 264.7. KYN treatment (10 μM or 25 μM) for 48 h does not affect viability of Raw 264.7 cells either alone (**A**) or with co-treatment of RANKL (**B**). Data are presented as mean ± SD (*n* = 6–12). Experiments were independently repeated three times for each condition, and a one-way ANOVA test was applied. (**C**) Representative images of TRAP stained Raw 264.7 cells treated with KYN at concentration of 10 μM or 25 μM for 5 days with no RANKL (upper panel) or with RANKL addition (lower panel). (**D**) The total number of TRAP+ multinucleated cells per well in each condition. Data are presented as mean ± SD (*n* = 4–6). Experiments were independently repeated three times for each condition, and a one-way ANOVA test was applied. Images were taken at 10X objective magnification with scale bar indicated. TRAP+ multinucleated cells were counted blindly.

The cytotoxic effect of Kynurenine (KYN) treatment was tested at doses of 10 and 25 μM on Raw 264.7 cells in presence or absence of RANKL. Neither of the doses altered Raw 264.7 cells viability (Figure 1A,B). The enzyme tartrate-resistant acid phosphatase (TRAP) is used as a histochemical marker to identify multinucleated osteoclastic cells. We treated Raw 264.7 cells with different doses of KYN (10 μM or 25 μM) either alone (served as controls) or with RANKL for 5 days and then stained for TRAP (Figure 1C). Treatment with KYN significantly enhanced RANKL-dependent osteoclastogenesis in a dose-dependent manner. The number of mature osteoclasts (TRAP + cells with three or more nuclei) were significantly increased when treated with KYN (10 μM or 25 μM) in the presence of RANKL compared to the RANKL-only controls (Figure 1D). Treatment with KYN alone did not induce osteoclast differentiation (Figure 1C).

### 2.2. Kynurenine Treatment Induces RANKL-Mediated Osteoclast Bone Resorption Activity of Raw 264.7 Cells

**Figure 2:**
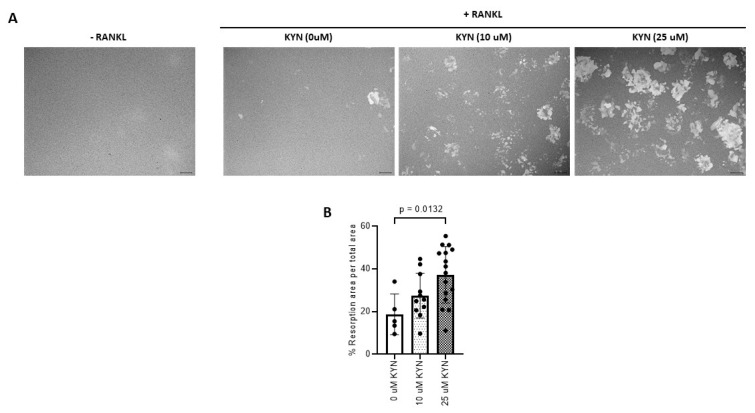
Kynurenine (KYN) treatment induces RANKL-mediated osteoclast bone resorption activity of Raw 264.7 cells. (**A**) Representative images of resorption lacunae formed when an equal number of Raw 264.7 cells were seeded with KYN at concentrations of 10 or 25 μM for 48 h without RANKL addition (Left panel) or with RANKL (Right panel). (**B**) The percentage of resorption area per total area in each condition. Data are presented as mean ± SD. Experiments were independently repeated two times for each condition. ANOVA analysis was used. Images were taken at 10X objective magnification with scale bar indicated.

Using Corning Osteo Assay surface plates, we found that KYN treatment significantly increased the bone resorptive activity of RANKL-induced Raw 264.7 osteoclastic cells (Figure 2A). The % of resorption area formed by active osteoclasts in KYN/RANKL-treated cells was significantly higher when compared to % resorption area formed by RANKL-only stimulation (Figure 2B).

### 2.3. Kynurenine Treatment Upregulates the Expression of Osteoclast Differentiation Markers c-fos and NFATc1

**Figure 3:**
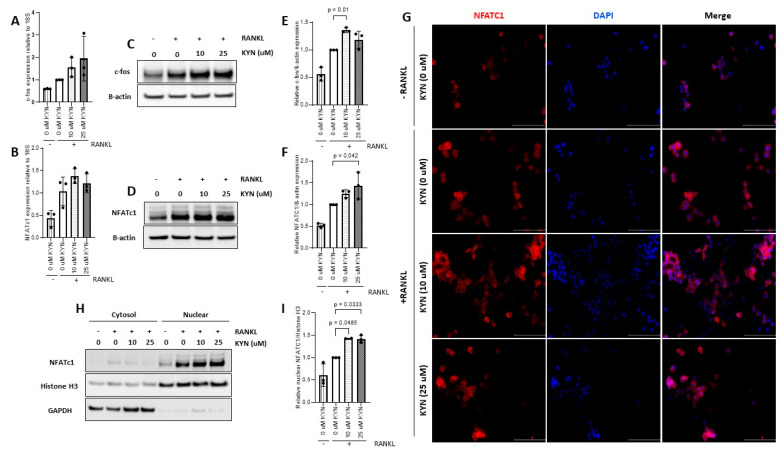
Kynurenine (KYN) treatment upregulates the expression of osteoclast differentiation markers c-fos and NFATc1. mRNA expression level of c-fos (**A**) or NFATc1 (**B**) in Raw 264.7 cells treated with KYN (10 or 25 μM) in presence of RANKL for 24 h. Data are presented relative to RANKL-only (0 μM KYN) control. Data are representative of three independent experiments and presented as mean ± SD. One-way ANOVA test was applied. Representative Western blotting of c-fos (**C**) or NFATc1 (**D**) proteins in Raw 264.7 cells treated with KYN (10 or 25 μM) in presence of RANKL for 24 h. (**E**) Densitometric analysis of c-fos protein expression in (**C**). (**F**) Densitometric analysis of NFATc1 protein expression in (**D**). Data are expressed as c-fos/β-actin or NFATc1/β-actin ratio relative to RANKL-only (0 μM KYN) control. Data are representative of three independent experiments and presented as mean ± SD. One-way ANOVA test was applied. (**G**) Immunocytochemistry analysis of Raw 264.7 cells treated with KYN (10 μM or 25 μM) in presence of RANKL for 24 h using NFATc1 (red) antibody. Nuclei were visualized using DAPI (blue). Images are representative of two independent experiments. Images were taken at 40X objective magnification with scale bar indicated. (**H**) Representative Western blotting of cytoplasmic and nuclear fractions of NFATc1 in Raw 264.7 cells treated with KYN (10 or 25 μM) in the presence of RANKL for 24 h. Histone H3 was used as nuclear marker and GAPDH was used as cytoplasmic marker. (**I**) Densitometric analysis of nuclear NFATc1 localization. Data are expressed as NFATc1/Histone H3 ratio relative to RANKL-only (0 μM KYN) control. Data are representative of three independent experiments and presented as mean ± SD. A one-way ANOVA test was applied.

c-fos and NFATc1 are key transcription factor regulators of the osteoclast differentiation process [24]. Accordingly, we tested the effect of KYN treatment on expression levels of NFATc1 and c-fos. We found that KYN/RANKL treatment for 24 h increased the mRNA expression of c-fos and NFATc1 compared to RANKL-only treated cells (Figure 3A,B). These data were further confirmed by Western blotting analysis where we found that KYN/RANKL treatment for 24 h significantly increased the protein expression of c-fos and NFATc1 compared to RANKL-only treated cells (Figure 3C–F). Additionally, we examined the effect of KYN treatment on nuclear translocation of NFATc1. KYN co-treatment with RANKL (10 or 25 μM) increased nuclear translocation of NFATc1 as shown by significant increase in intensity and colocalization of NFATc1 (red) and DAPI (blue) in KYN-treated cells compared to RANKL-only treated cells (Figure 3G). This result was further confirmed by Western blot analysis of cytoplasmic and nuclear fractions of Raw cells treated with KYN/RANKL compared to RANKL-only treated cells (Figure 3H,I).

### 2.4. Blocking AhR Signaling Attenuates KYN/RANKL Induced Osteoclast Differentiation of Raw 264.7 Cells via Inhibiting c-fos and NFATc1 Expression

**Figure 4:**
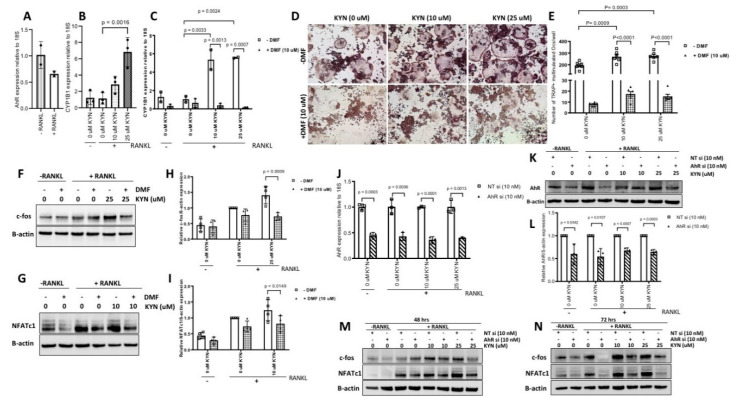
Blocking AhR signaling attenuates KYN/RANKL regulation of osteoclast differentiation in Raw 264.7 cells via inhibition of c-fos and NFATc1. (**A**) mRNA expression level of AhR in Raw 264.7 cells in the absence or presence of RANKL for 24 h. Data are presented relative to—RANKL control. Data are representative of two independent experiments and presented as mean ± SD. Unpaired t-test was applied. (**B**) mRNA expression level of CYP1B1 in Raw 264.7 cells treated with KYN (10 or 25 μM) in presence of RANKL for 24 h. Data are presented relative to RANKL-only (0 μM KYN) control. Data are representative of three independent experiments and presented as mean ± SD. A one-way ANOVA test was applied. (**C**) mRNA expression level of CYP1B1 in Raw 264.7 cells treated with RANKL/KYN at concentration of 10 μM or 25 μM KYN for 24 h in presence or absence of DMF (10 μM). Data are presented relative to RANKL-only (0 μM KYN) control. Data are representative of two independent experiments and presented as mean ± SD. A two-way ANOVA test was applied. (**D**) Representative images of TRAP stained Raw 264.7 cells treated with RANKL/KYN at concentration of 10 or 25 μM for 5 days with no DMF added (upper panel) or with DMF (10 μM) (lower panel). (**E**) The total number of TRAP+ multinucleated cells per well in each condition. Data are presented as mean ± SD (*n* = 5–6). Experiments were repeated three times for each condition. A two-way ANOVA test was applied. Images were taken at 10X objective magnification with scale bar indicated. TRAP+ multinucleated cells were counted blindly. (**F**,**G**) Representative Western blotting of c-fos and NFATc1 proteins expression in Raw 264.7 cells treated with RANKL/KYN (10 or 25 μM) for 24 h in the presence or absence of DMF (10 μM). (**H,I**) Densitometric analysis of c-fos and NFATc1 proteins expression in (**F**,**G**). Data are expressed as c-fos/β-actin or NFATc1/β-actin ratio relative to RANKL-only (0 μM KYN) control. Data are representative of three independent experiments and presented as mean ± SD. A two-way ANOVA test was applied. (**J**) mRNA expression level of AhR in Raw 264.7 cells transfected with NT siRNA or AhR siRNA before treating with RANKL/KYN (10 or 25 μM) and collected 48 h post-transfection. Data are presented relative to NT siRNA control for each condition. Unpaired t-test was applied. (**K**) Representative Western blotting of AhR protein expression in Raw 264.7 cells transfected with NT siRNA or AhR siRNA before treating with RANKL/KYN (10 or 25 μM) and collected 72 h post-transfection. (**L**) Densitometric analysis of AhR protein expression in (**K**). Data are expressed as AhR/β-actin relative to NT siRNA control for each condition. Unpaired t-test was applied. Data are representative of two independent experiments and presented as mean ± SD. (**M**,**N**) Representative Western blotting of c-fos and NFATc1 proteins expression in Raw 264.7 cells transfected with NT siRNA or AhR siRNA before treating with RANKL/KYN (10 or 25 μM) and collected 48 h (**M**) or 72 h (**N**) post-transfection. Data are representative of two independent experiments.

AhR is a known receptor for KYN [12]. We verified the expression of AhR in Raw 264.7 cells and we found that RANKL treatment caused a non-significant decrease in AhR mRNA levels (Figure 4A). Ligand activated AhR upregulates target genes such as CYP1B1, which has been implicated in bone resorption activity. To examine whether KYN treatment induces AhR signaling in Raw 264.7 cells, we treated Raw 264.7 cells with KYN/RANKL (10 or 25 μM) for 24 h and measured the change in expression level of the CYP1B1 gene. We found that KYN/RANKL treatment significantly increased the expression levels of CYP1B1 compared to RANKL-only control cells (Figure 4B). The increase in CYP1B1 gene expression was diminished when Raw 264.7 cells were pretreated with 10 μM 3′,4′-dimethoxyflavone (DMF) (an AhR antagonist, which acts as a competitive inhibitor) prior to treating with KYN/RANKL for 24 h (Figure 4C).

To test whether KYN treatment induces osteoclast differentiation via AhR signaling, we pretreated Raw 264.7 cells with DMF (10 μM) for 12 h before treating with or without KYN/RANKL for 5 d. At the end of day 5, cells were stained for TRAP and the number of TRAP+ multinucleated cells were counted. The results showed that blocking AhR with DMF abolished KYN/RANKL induced osteoclast differentiation (Figure 4D). The high number of TRAP+ multinucleated cells formed as result of KYN/RANKL treatment was significantly reduced upon blocking AhR signaling (Figure 4E). This suggests that AhR signaling is regulating KYN’s effects on the increase of RANKL-mediated osteoclast formation. Recently, it has been reported that AhR signaling might be regulating the expression of NFATc1, but not c-fos. We examined the effect of AhR blocking on c-fos and NFATc1 expression as key regulators of osteoclast differentiation. We found that blocking AhR with 10 μM DMF reduced the KYN/RANKL induced upregulation of both c-fos and NFATc1 levels (Figure 4F–I). To confirm this result, genetic silencing of AhR was performed (Figure 4J–L). Similar to the use of the AhR inhibitor DMF, the genetic knock down of AhR abolished the KYN/RANKL induced upregulation of c-fos and NFATc1 protein levels (Figure 4M,N). These data suggest that KYN is inducing osteoclastogenesis via the AhR signaling pathway.

## 3. Discussion

Aging is a degenerative process that is associated with a decrease in bone mass and the development of aging-related bone disorders such as osteoporosis and increased risk for bone fractures and their potential life-altering sequala [25,26]. Several studies have shown that kynurenine (KYN), a metabolite that can be enzymatically or oxidatively derived from tryptophan, accumulates with aging, may have a role in bone remodeling process [11,12]. Normal physiological bone remodeling is maintained by proper balance and communication between two main processes: osteogenesis (bone formation) and osteoclastogenesis (bone resorption) [27]. Bone remodeling is a complex process that is controlled by many local and systemic factors. This includes calcitonin, parathyroid hormone, vitamin D3 and estrogen, among others [28]. Pathological conditions as osteoporosis or aging lead to disruption of the balance between bone formation and bone resorption. As we age, factors deriving osteoclast differentiation and activity increases; however, the amount of bone formed with each cycle of bone remodeling decreases [4]. To restore the balance between bone formation and bone resorption, strategies to induce osteoblast formation and activity or reduce osteoclast differentiation and activity are now of great interest [29]. It has been shown by our group that KYN induces bone loss by suppressing osteogenesis and increasing osteoclastogenesis [11,12,14]. It has also been shown that chronic kidney disease-associated accumulation of peripheral KYN might contribute to the decrease in bone formation and increase in the bone resorption observed in chronic kidney disease [30]. To date, the direct effect of KYN on osteoclastogenesis has not been studied. Here, we investigate the direct effect of KYN treatment on receptor activator of nuclear factor kappa B ligand (RANKL)-induced in vitro osteoclastogenesis. We aimed to understand the molecular mechanisms by which KYN might be inducing osteoclast formation and hence presenting a potential therapeutic target that might be useful in the prevention or treatment of aging or disease-associated bone loss with additional studies.

Osteoclasts are large multinucleated bone resorbing cells that differentiate from monocyte/macrophage precursor cells under the regulation of RANK/RANKL signaling. Activation of RANK/RANKL signaling pathway induces downstream signaling cascades that upregulate the expression of specific genes involved in osteoclast differentiation, activation and survival [22,31]. c-fos and NFATc1 are key transcription factors for RANKL-induced osteoclast differentiation [32,33]. Several in vivo and in vitro studies have established the role of c-fos and NFATc1 in osteoclast differentiation [34,35,36]. Downstream target genes that are regulated by NFATc1, include TRAP, CTSK, DC-STAMP and OC-STAMP [31,33,37,38]. Accordingly, we tested the effect of KYN treatment on RANKL-induced osteoclast differentiation in Raw 264.7 cells. Our results showed that RANKL treatment alone induced osteoclast formation as evident by formation of large multinucleated TRAP+ cells. Combining RANKL with KYN treatment significantly increased the number of mature osteoclasts formed. However, KYN treatment alone was insufficient to induce osteoclast formation. Similarly, RANKL/KYN treatment significantly increased the bone resorption activity of differentiated osteoclast as compared to RANKL-only treatment. Consistently, it has been reported that IL-35 [39], monosodium urate [40] and succinate [41] enhanced osteoclastogenesis when combined with RANKL, similar to KYN without RANKL co-treatment they were not able to induce osteoclastogenesis. Although KYN treatment alone slightly upregulated c-fos and NFATc1 expression (unpublished data), the increase was insufficient to induce ostoeclastogenesis. We suggest that KYN treatment accelerates or amplifies osteoclastogenesis in the presence of RANKL, which is required to drive osteoclast differentiation and activation. We propose that the physiological role KYN plays in inflammation is critical in bone loss. Macrophages/Osteoclasts strongly express IDO-1 and IDO-1 expression in BMSC’s is increased by inflammation (e.g., Interferon gamma) [11]. As such, we view KYN as a nutrient mediator generated by the macrophages to promote their differentiation to mature osteoclasts in the setting of age-related inflammation.

To investigate how KYN increases RANKL-dependent osteoclastogenesis, we measured the changes in expression of c-fos and NFATc1. qRT-PCR, cellular fractionation, Western blotting and immunofluorescence analyses showed that KYN/RANKL treatment upregulates mRNA and protein expression levels of c-fos and NFATc1 and induced higher nuclear localization of NFATc1 when compared to RANKL-only treatment. This supports the idea that the higher number of TRAP+ multinucleated cells formation observed upon treating Raw 264.7 cells with KYN/RANKL compared to RANKL-only stimulation was due to the upregulation of these transcription factor pathways. These results suggest that KYN acts to increase of RANKL-dependent osteoclastogenesis, which is consistent with our previous study showing an increase in osteoclast numbers and activity in mice injected or fed with KYN [11].

**Figure 5:**
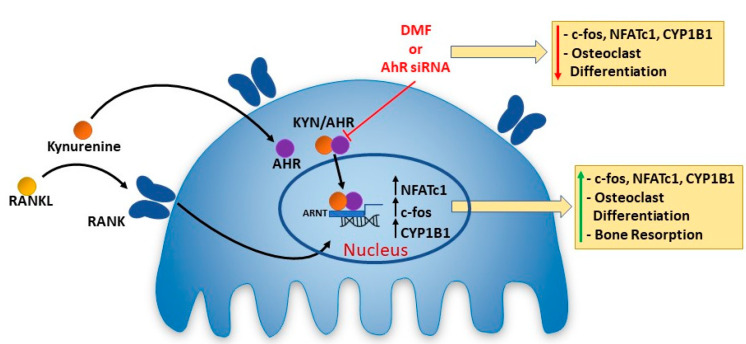
Kynurenine Induces Osteoclastogenesis Via the AhR Signaling Pathway. In the presence of RANKL, KYN induces Raw 264.7 cells to undergo osteoclastogenesis. This is mediated by KYN binding to AhR which translocates to the nucleus. Based on the literature the ligand bound to AhR forms a transcription factor complex with ARNT [12]. This complex binds to the Xenobiotic responsive elements in the promotor regions of target genes, which here include c-fos, NFATc1 and CYP1B1 [43,45,46,47]. The down-stream consequence is osteoclast differentiation and activation of osteoclast bone resorption. These effects can be blocked by pharmacologic inhibition of AhR with the DMF, or genetic inhibition with AhR siRNA. This suggests that inhibiting KYN levels, blocking KYN binding of AhR, or blocking KYN/AhR signaling may be potential therapeutic approaches to limit the role of osteoclast activity in age-associated bone loss or osteoporosis.

Several studies have reported a contradictory role for AhR signaling in osteoclastogenesis [22]. This may be due to distinct ligands activating AhR signaling in diverse ways, e.g., due to differences in co-factor recruitment and assembly resulting in the targeting of unique sets of genes for expression, resulting in the induction, or inhibition, of osteoclast differentiation and resorptive activity [17,18,21,42,43]. KYN is an AhR ligand that upon binding induces AhR transport to the nucleus and subsequent association with ARNT in forming a transcription factor complex leading to transcriptional activity of target genes including CYP1A1 and CYP1B1 via binding to the xenobiotic response elements [44]. We first confirmed activation of AhR signaling in Raw 264.7 cells upon treatment with KYN/RANKL. Our results show that KYN/RANKL treatment significantly upregulated the mRNA expression of the target gene CYP1B1. Importantly, blocking AhR signaling using the AhR competitive inhibitor DMF, significantly reduced the KYN/RANKL upregulation of CYP1B1 mRNA. In addition, our results show that inhibiting AhR signaling using DMF, an AhR antagonist, significantly attenuated the KYN/RANKL mediated increase in osteoclast differentiation. The high number of TRAP+ multinucleated cells formed by KYN/RANKL stimulation of Raw 264.7 cells was significantly reduced upon blocking AhR signaling. These data suggest that KYN enhances RANKL-induced osteoclastogenesis via activating the AhR signaling pathway (Figure 5). These findings are consistent with Kalaska et al., who reported that elevated peripheral KYN levels in nephrectomy induced-chronic kidney disease reduced bone mass via activating the AhR pathway [30].

As shown in Figure 3, KYN/RANKL treatment enhanced mRNA and protein expression levels of both c-fos and NFATc1. To test whether KYN/RANKL upregulation of c-fos and NFATc1 was mediated by AhR signaling, we investigated the differential expression of c-fos and NFATc1 upon blocking AhR signaling. Indeed, we found that KYN/RANKL induced upregulation of c-fos and NFATc1 was significantly reduced upon blocking AhR signaling using DMF. To confirm these data, we used AhR siRNA, and an even more pronounced reduction of c-fos and NFATc1 proteins expression was observed. These results suggest that AhR is acting as an upstream regulator of c-fos and NFATc1. These findings, in part, agreed with Liu et al., who reported that Indoxyl Sulfate activates NFATc1, but not c-fos, via AhR signaling pathway [48]. It has been also reported that activating AhR signaling by benzo[a]pyrene ligand induces osteoclast differentiation via upregulating c-fos and NFATc1 expression [43,49]. Consistent with the contradictory role of AhR in osteoclastogenesis, AhR regulation of c-fos and NFATc1 expression is also a matter of debate. A study by Mejía-García et al., reported that 2,3,7,8-Tetrachlorodibenzo-p-dioxin (TCDD) and β-naphthoflavone (β-NF)-mediated AhR activation induced mRNA and protein expression of Ubcm4 ubiquitin-conjugating enzyme. This resulted in ubiquitination and proteasome degradation of proteins including c-fos and others [50]. Their study, however, did not address whether AhR-mediated c-fos ubiquitination was an AhR-ligand specific phenomenon. Another study by Talari et al., reported a negative correlation between AhR and c-fos expression levels in human meningioma [51]. On the contrary, Tang et al., reported an AhR-dependent activation of c-fos expression in dioxin-exposed humans with chloracne skin disease [52]. The variation in AhR regulation of c-fos and NFATc1 among different studies might be due to difference in the specific ligand used to activate AhR signaling, the duration of AhR-ligand binding and different transcription complex components, and the difference in the biological model used to study the AhR/c-fos/NFATc1 axis.

In conclusion, we show that KYN increases osteoclastogenesis and we provide for the first time a potential mechanism by which KYN might be acting on pre-osteoclastic and osteoclastic cells (Figure 5). We show that KYN enhances and drives RANKL-induced osteoclast differentiation and resorption activity via the AhR signaling pathway, which upregulates c-fos and NFATc1, the key transcription factors in osteoclast formation (Figure 5). While a recent in vivo study by our group has shown that KYN induces bone loss via increasing osteoclastic activity [11], more studies are needed to further establish KYN-AhR mediated enhancement of osteoclast differentiation. Altogether, the findings of this study are providing a step forward in understanding the role of KYN in osteoclastogenesis. As such the KYN-AhR system is a novel research avenue that needs further investigation to address bone loss in aging-associated diseases including osteoporosis.

## 4. Materials and Methods

### 4.1. Cell Culture

Raw 264.7 Murine macrophage cells (ATCC TIB-71TM, Manasas, VA, USA) were cultured and maintained in Dulbecco’s modified Eagle’s medium (DMEM) (#10-014-CV, Corning, NY, USA) supplemented with 10% heat-inactivated fetal bovine serum (FBS) (#S11150 Atlanta Biologicals, R&D Systems, Minneapolis, MN, USA) and 1X of Antibiotic/Antimycotic Solution (#SV30079.01, HyClone Logan, UT,USA) in a humidified 5% CO_2_ incubator at 37 °C (Forma Series II Water Jacketed CO_2_ incubator, ThermoFisher Scientific, Waltham, MA, USA).

### 4.2. Kynurenine (KYN) Doses and Cell Viability Assay

Kynurenine (KYN) (#K8625, Sigma-Aldrich, St. Louis, MO, USA) treatment used in this study at a concentration of 10 and 25 μM. The selection of these doses was based on preliminary experiments as well as data reported previously in the literature. KYN levels in different culturing systems are highly variable and range from 5–60 μM [11,14,15,53,54,55]. As the DMEM media itself contains 3–5 μM KYN [12], we therefore used 10 μM as the lowest dose for in vitro treatment as well as 25 μM as an intermediate KYN dose since those are close to the concentrations seen in numerous cells and tissue microenvironments.

The effect of KYN treatment on Raw 264.7 cells viability was assessed using AlamarBlue HS Cell Viability Reagent (#A50100, Thermo Fisher Scientific, Waltham, MA, USA) according to manufacturer’s instructions. In brief, Raw 264.7 cells (5 × 10^3^ cells/well) were seeded in 96 well plates in DMEM complete media supplemented with or without 100 ng/mL RANKL (recombinant murine soluble Receptor Activator of Nuclear Factor-κB Ligand) (# 315-11, PeproTech, Cranbury, NJ, USA), and treated with or without KYN (10 μM or 25 μM) for 48 h. Then media was removed and replaced with DMEM phenol red free media (#11054020, Thermo Fisher Scientific, Waltham, MA, USA) mixed with AlamarBlue HS Cell Viability Reagent and cells were re-incubated for 2.5 h at 37 °C in 5% CO_2_ incubator protected from light. The absorbance of intense red color formed as a result of cellular reduction of resazurin to resorufin was measured at 570 nm using 600 nm as a reference wavelength using SUNRISE spectrophotometer (TECAN, Morrisville, NC, USA).

### 4.3. Osteoclast Differentiation and Tartrate-Resistant Acid Phosphatase (TRAP) Staining

For osteoclast differentiation, 5 × 10^3^ Raw 264.7 cells/well were cultured in 96 well plate in complete DMEM media supplemented with 100 ng/mL of RANKL. To test the effect of KYN treatment on osteoclast differentiation, cells were treated with or without KYN (10 or 25 μM) and kept in a humidified 5% CO2 incubator at 37 °C for 5 d with media being replaced every other day. Multinucleated cells started to appear at day 3. On day 5, numerous mature osteoclasts with three or more nuclei were formed. At the end of day 5, cells were fixed and stained following instructions of Acid Phosphatase, Leukocyte (TRAP) staining kit (# 387, Sigma-Aldrich, St. Louis, MO, USA). Four images per well, covering all well surface, were taken using ImageXpress Micro High Content Imager (Molecular Device, San Jose, CA, USA). TRAP-positive cells with 3 or more nuclei were blindly counted as osteoclasts. Representative images for each well were taken with a Keyence BZ-X710 microscope (Keyence Corporation Of America, Itasca, IL, USA) at 100X magnification.

### 4.4. Hydroxyapatite Resorption Assay

The effect of KYN treatment on osteoclast’s resorption activity was tested using Corning Osteo Assay Surface (#3998, Corning Lifesciences, Corning, NY, USA). Briefly, Raw 264.7 cells at density of 5 × 10^3^ cells/well were seeded in plastic 96 well plate for 4 d with or without 100 ng/mL RANKL and KYN (10 or 25 μM) with media change every other day. Then, mature osteoclasts were collected and counted. Equal number of mature osteoclasts were seeded in Corning Osteo Assay Surface plate and further treated KYN (10 or 25 μM) for an additional 48 h. At end of day 6, media was carefully aspirated, and 10% bleach solution was added, and plate was incubated for 5 min at room temperature. Wells were washed twice with distilled water and the plate was allowed to air dry. The plate was visualized, and images were taken using Keyence BZ-X700 microscope at 100X magnification. The percentage of resorption area was blindly analyzed using Image J 1.48v image analysis software (National Institutes of Health, Bethesda, MD, USA).

### 4.5. Western Blotting

Total lysates of Raw 264.7 cells collected from different experiments were prepared using RIPA lysis and extraction buffer (#89901, ThermoFisher Scientific, Waltham, MA, USA) to which cOmplete™ Protease Inhibitor Cocktail (#11697498001, Millipore Sigma, St. Louis, MO, USA) and PhosSTOP phosphatase inhibitor (#4906845001, Millipore Sigma, St. Louis, MO, USA) were freshly added. Extraction of separate cytoplasmic and nuclear protein fractions were prepared using NE-PER™ Nuclear and Cytoplasmic Extraction Reagents (#78833, ThermoFisher Scientific, Waltham, MA, USA) to which cOmplete™ Protease Inhibitor Cocktail was added. Protein concentrations were measured using Pierce BCA Protein Assay Kit (#23225, ThermoFisher Scientific, Waltham, MA, USA). Equal amounts of protein lysates (20 μg) were separated using gradient 4–12% NuPAGE Bis-Tris gels and transferred to nitrocellulose membranes using Power Blotter pre-cut membranes and filters (#PB7320, ThermoFisher Scientific, Waltham, MA, USA). Following transfer, membranes were blocked for 1 h using 5% Blotting Grade Blocker Non-Fat Dry Milk (#1706404, Bio-Rad, Hercules, CA, USA). Membranes were incubated with appropriate primary antibody overnight at 4 °C. Primary antibodies NFATc1 (#sc-7294), c-fos (#sc-166940) (Santa Cruz Biotechnology, Dallas, TX, USA), AhR (#AF6697, R&D Systems, Minneapolis, MN, USA), Histone H3 (#4499T) and GAPDH (#2218S) (Cell signaling, Danvers, MA, USA) and ß-actin (#A5441, Millipore sigma, St. Louis, MO, USA) were used. Membranes were washed 3 times for 10–15 min and then incubated with appropriate HRP-conjugated secondary antibody for 1 h at room temperature. Membranes were washed 3 times for 10–15 min and visualized using Pierce ECL detection system (#32106, ThermoFisher Scientific, Waltham, MA, USA) on Amersham Imager 600 (GE Healthcare, Pittsburgh, PA, USA). The intensity of immunoreactive bands was quantified using Image Lab (Bio-Rad, Hercules, CA, USA).

### 4.6. Quantitative Real-Time Polymerase Chain Reaction (qRT-PCR)

Total RNA of Raw 264.7 cells collected from different experiments was extracted using RNAeasy kit (#74106 Qiagen, Santa Clarita, CA, USA). Complementary DNA (cDNA) was synthesized using High-Capacity cDNA Reverse Transcription Kit (#4368814, Applied Biosystems, Waltham, MA, USA) following the manufacturer’s instructions. PCR reactions were performed using TaqMan Fast Advanced Master Mix (#4444556, Applied Biosystems, Waltham, MA, USA). The pre-formulated assay primers (#4331182, Applied Biosystems, Waltham, MA, USA) used in this study are listed in Table 1. Gene expression levels were quantified using the comparative threshold cycle (2^-∆∆^Ct) method. 18S was used as housekeeping gene control for normalization of target gene expression levels.

### 4.7. Immunofluorescence Microscopy

Raw 264.7 cells were seeded in eight-well chamber slides (Nunc™ Lab-Tek™ II Chamber Slide™ System, # 154534, Thermo Fisher Scientific, Waltham, MA, USA) and treated with RANKL 20 ng/mL with or without KYN (10 or 25 μM) for 24 h to check NFATc1 nuclear translocation. Cells were fixed in 4% paraformaldehyde/PBS solution for 30 min at room temperature. Cells were washed with PBS and permeabilized with 0.1% Triron-X-100/PBS for 5 min at room temperature. Cells were washed with PBS and blocked with 10% donkey serum overnight at 4 °C. Cells were incubated with NFATc1 (#sc-7294) followed by donkey anti-mouse IgG Alexa Fluor 594-conjugate (#A32744, Thermo Fisher Scientific, Waltham, MA, USA). Slides were mounted using ProLong™ Diamond Antifade Mountant with DAPI to stain nuclei (#P36962, Thermo Fisher Scientific, Waltham, MA, USA). Images were captured using Keyence BZ-X700 microscope at 400X magnification.

### 4.8. AhR Blocking

To examine the effect of AhR blocking on RANKL/KYN- induced osteoclastogenesis, Raw 264.7 cells, were pre-treated for 12 h with an AhR antagonist 3′,4′-dimethoxyflavone (DMF) (#D6571, Sigma-Aldrich, St. Louis, MO, USA) at final concentration of 10 μM. Following pre-treatment, Raw 264.7 cells were induced for osteoclast differentiation using 100 ng/mL RANKL with or without KYN (10 or 25 μM) for 5 days. At the end of day 5, TRAP staining was performed as described above.

The effect of AhR signaling blocking on c-fos and NFATc1 expression regulation was tested by pre-treating Raw 264.7 cells with 10 μM DMF for 12 h followed by treating the cells with 20 ng/mL RANKL with or without KYN (10 or 25 μM) for an additional 24 h. Cells were collected and used for further Western blot or qRT-PCR analyses.

### 4.9. AhR Silencing

To confirm the effect of blocking AhR signaling on regulating c-fos and NFATc1 expression, genetic knock down of AhR was performed. In Brief, Raw 264.7 cells at density of 4 × 10^4^ cells/well were seeded in 12 well plate and allowed to attach overnight. Next day, cells were transfected using Viromer Blue transfection reagent (#TT100300, OriGene Technologies, Rockville, MD, USA) and Ahr Mouse Small interfering RNA (siRNA) Oligo Duplex (AhR si, # SR420301C, OriGene Technologies, Rockville, MD, USA) at a final concentration of 10 nM. Trilencer-27 Universal scrambled negative control siRNA duplex was used to transfect control groups at a final concentration of 10 nM. One or two days after transfection, cells were treated with KYN (10 or 25 μM) with or without RANKL (20 ng/mL) for 24 h before collecting the cells for further Western blotting analyses.

### 4.10. Statistical Analysis

All experiments were repeated independently at different times at least three times, unless stated otherwise. GraphPad Prism version 8.3.0 for Windows (GraphPad Software, San Diego, CA, USA) was used to perform the statistical analyses. Data are presented as mean ± standard deviation (SD). Two groups were compared using unpaired t-test. Three or more groups were compared using one-way or two-way analysis of variance (ANOVA) followed by Tukey’s or Sidak’s multiple comparison post-hoc tests, respectively. Values of *p* < 0.05 were considered statistically significant.

## Figures and Tables

**Table 1:** TaqMan mRNA pre-formulated gene specific primer assay IDs.

Gene	Assay ID
18S	Mm03928990_g1
AhR	Mm00478932_m1
c-fos	Mm00487425_m1
NFATc1	Mm01265944_m1
CYP1B1	Mm00487229_m1

## Abbreviations

AhR	Aryl hydrocarbon receptor
BaP	Benzo(a)pyrene
BMSCs	Bone marrow mesenchymal stem cells
Cyp1A/1B	cytochrome P450 1a/1b
DMEM	Dulbecco’s modified Eagle’s medium
DMF	3′,4′-dimethoxyflavone
FBS	Heat-inactivated fetal bovine serum
IDO-1 or 2	Indoleamine 2,3-dioxygenase-1 or 2
KYN	kynurenine
RANKL	Receptor activator of nuclear factor kappa-B ligand
siRNA	Small interfering RNA
TCDD	2,3,7,8-tetrachlorodibenzo-p-dioxin
TDO	Tryptophan 2,3-dioxygenase
TRAP	Tartrate-resistant acid phosphatase
β-NF	β-naphthoflavone
